# Oncolytic virus therapy for osteosarcoma: mechanisms, opportunities, and challenges

**DOI:** 10.3389/fimmu.2025.1724768

**Published:** 2025-12-10

**Authors:** Jia-Wen Wang, Xiaolei Ge, Jia-Hui Liu, Xiaoyu Zhang

**Affiliations:** 1 Department of Orthopedics, The Fourth Hospital of Hebei Medical University, Shijiazhuang, Hebei, China; 2 Wangjing Hospital of the Chinese Academy of Chinese Medical Sciences, Beijing, China

**Keywords:** oncolytic virus, osteosarcoma, immunogenic cell death, tumor microenvironment, precision medicine

## Abstract

Osteosarcoma (OS), the most common primary malignant bone tumor in children and adolescents, remains a major therapeutic challenge due to its high metastatic potential and limited response to conventional treatments, including immune checkpoint inhibitors (ICIs). Oncolytic viruses (OVs) have emerged as a promising strategy with dual antitumor functions: direct oncolysis and the induction of immunogenic cell death (ICD). By releasing damage-associated molecular patterns (DAMPs) and activating the cGAS–STING pathway, OVs can remodel the immunologically “cold” tumor microenvironment (TME) into an inflamed and immune-responsive phenotype, thereby enhancing CD8^+^ T-cell infiltration and improving antitumor immunity. Encouraging preclinical evidence has been reported: VSV-IFNβ-NIS achieved a long-term survival rate of approximately 35% in canine OS models, and synergistic combination regimens have demonstrated tumor inhibition rates exceeding 70%. Despite these advances, OV-based therapies still face critical translational challenges, including the immunosuppressive TME, intratumoral delivery barriers, and safety concerns. This review systematically summarizes the molecular mechanisms underlying OV-mediated antitumor immunity, evaluates current clinical evidence, and highlights future opportunities, such as combination immunotherapy, mesenchymal stem cell (MSC)-based delivery platforms, and AI-driven precision medicine approaches. Our goal is to provide a comprehensive theoretical framework to support the clinical translation and personalized application of OV therapy in osteosarcoma.

## 1 Introduction

Osteosarcoma (OS) is the most common primary malignant bone tumor, predominantly affecting children and adolescents. It is characterized by high aggressiveness and a strong propensity for metastasis, particularly to the lungs, which remains the leading cause of mortality (1). While neoadjuvant chemotherapy combined with surgical resection can increase the 5-year survival rate to approximately 70% for patients without metastasis, this rate drops by more than half once pulmonary metastasis develops (1). Even with multidrug chemotherapy, the 5-year survival rate remains below 20%, especially in cases where metastatic lesions cannot be completely removed (2, 3). Moreover, conventional immunotherapies such as PD-1/PD-L1 inhibitors have demonstrated limited clinical benefit in OS, largely due to its inherent low tumor mutational burden (TMB), impaired antigen presentation, and profoundly immunosuppressive tumor microenvironment (TME), leading to objective response rates typically below 10% (4, 5). These limitations underscore the urgent need for novel therapeutic approaches capable of reshaping OS immunogenicity and improving survival outcomes.

Oncolytic viruses (OVs) have emerged as a promising immunotherapeutic strategy for OS (6), offering dual antitumor mechanisms. First, OVs selectively infect and lyse tumor cells, inducing immunogenic cell death (ICD) and releasing damage-associated molecular patterns (DAMPs), including ATP, HMGB1, and calreticulin, alongside tumor-associated antigens (TAAs), thereby mediating direct tumor cytotoxicity (7, 8). Second, OVs activate innate immune pathways, notably the cGAS-STING signaling axis, stimulating robust type I interferon responses and enhancing recruitment and activation of immune effector cells (9, 10). Concomitantly, OVs promote dendritic cell antigen presentation, trigger tumor-specific T-cell immunity, and drive macrophage polarization from an immunosuppressive M2 phenotype toward a pro-inflammatory M1 state, effectively converting an immunologically “cold” TME into a “hot” one enriched with infiltrating CD8^+^ T cells (11, 12). Recent findings further indicate that oHSV engineered to express GM-CSF can augment PD-1 blockade responses and strengthen NK-cell-mediated cytotoxicity, collectively reversing OS-associated immune suppression (13, 14).

Several OVs have already demonstrated clinical potential. T-VEC is FDA-approved for melanoma, and DNX-2401 has shown durable survival benefit and favorable safety in glioblastoma patients (15, 16). In the context of OS, agents such as VCN-01, Delta-24-ACT, and VSV-IFNβ-NIS have significantly suppressed tumor growth and extended survival in preclinical models (6, 17), offering a compelling foundation for further translational development.

**Figure 1:**
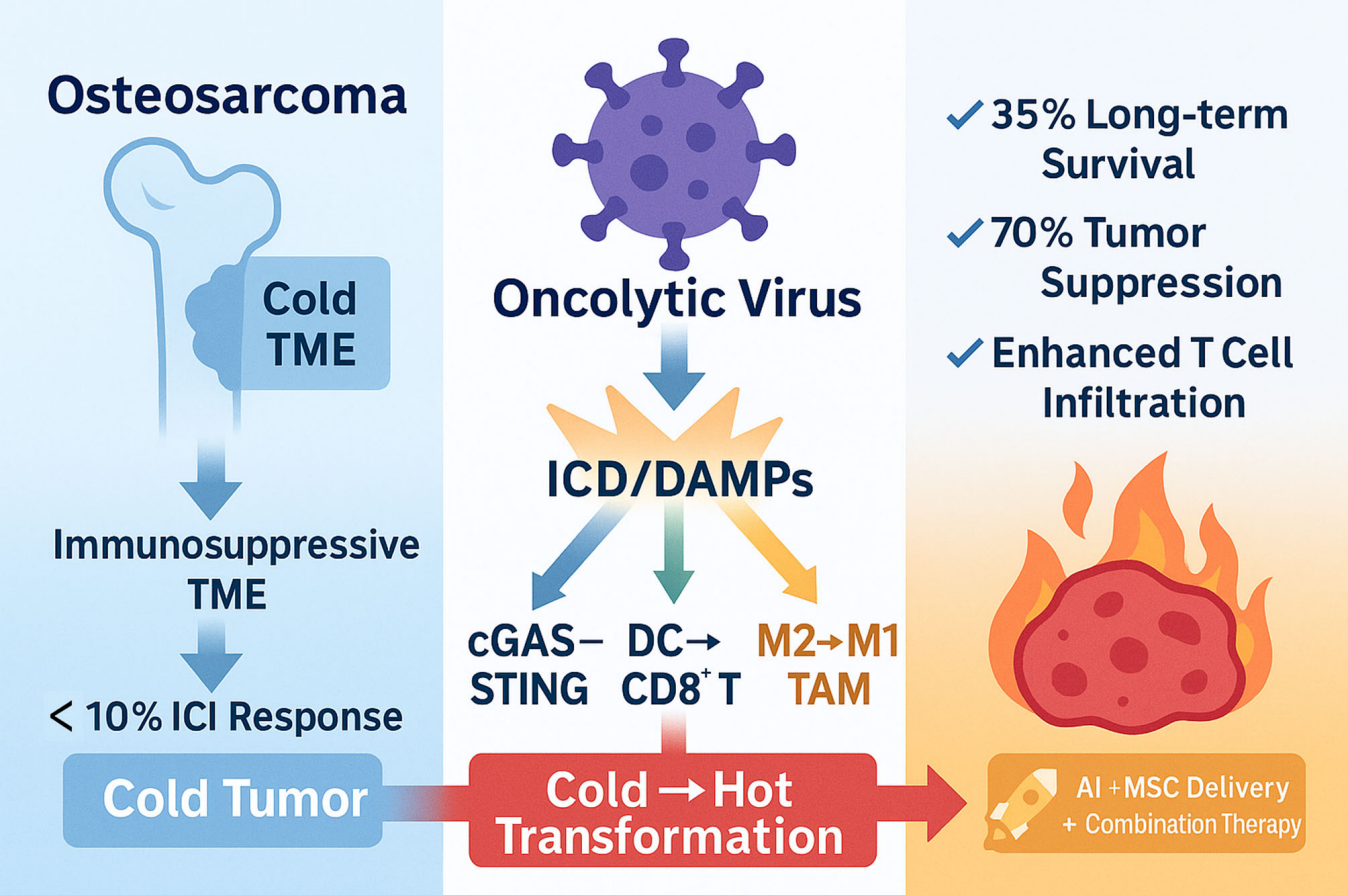
Oncolytic virus therapy for osteosarcoma: transforming “cold” into “hot” tumors. This figure illustrates the therapeutic paradigm of oncolytic virus (OV)-mediated immune remodeling in osteosarcoma. Left panel (Problem): Osteosarcoma exhibits an immunologically “cold” tumor microenvironment (TME) characterized by immunosuppression, resulting in minimal response to immune checkpoint inhibitors (ICIs; <10% objective response rate). Center panel (Mechanism): OVs selectively infect and lyse tumor cells, inducing immunogenic cell death (ICD) and releasing damage-associated molecular patterns (DAMPs). This triggers three parallel immune activation pathways: (1) cGAS-STING signaling (blue arrow) promotes type I interferon production; (2) Dendritic cell (DC) activation (green arrow) enhances CD8^+^ T cell priming and cross-presentation; (3) Tumor-associated macrophage (TAM) reprogramming (orange arrow) drives M2-to-M1 polarization with enhanced pro-inflammatory function. These coordinated mechanisms converge to transform immunologically “cold” tumors into “hot” immune-reactive environments. Right panel (Outcomes and Future): Preclinical and early clinical evidence demonstrates that OV therapy achieves 35% long-term survival rates and 70% tumor suppression, accompanied by enhanced T cell infiltration (represented by the inflamed tumor). Future translational strategies include artificial intelligence (AI)-guided precision medicine, mesenchymal stem cell (MSC)-based delivery systems to overcome delivery barriers, and rationally designed combination therapies to maximize therapeutic efficacy. Arrows indicate directional biological processes; color-coded pathways distinguish immune activation mechanisms; gradient transitions represent the cold-to-hot tumor transformation continuum.

This review aims to address four key questions (**Figure 1**):

1. How do OVs synergistically exert antitumor effects in OS through induction of ICD, activation of critical signaling pathways, and remodeling of the TME?
2. What is the current preclinical and clinical evidence regarding the efficacy and safety of OV-based therapies in OS, and what major obstacles—such as immune evasion and delivery barriers—impede clinical translation?
3. How can combination therapies with ICIs, novel delivery platforms, and optimized dosing strategies overcome these challenges and achieve superior therapeutic outcomes?
4. How might AI-driven personalized approaches and intelligent delivery systems advance OV therapy toward precision oncology in OS?

## 2 Core mechanisms of oncolytic virus therapy in osteosarcoma

Upon infection of tumor cells, oncolytic viruses (OVs) can induce immunogenic cell death (ICD) in osteosarcoma through multiple coordinated pathways, including caspase-3/7–dependent apoptosis, MLKL-mediated necroptosis, GSDME-dependent pyroptosis, and autophagy-associated cell death (18). These processes lead to the release of classical damage-associated molecular patterns (DAMPs), such as HMGB1, calreticulin, and ATP (19, 20), and promote the secretion of pro-inflammatory cytokines including IL-1β and IL-18 via GSDME-driven pyroptosis, thereby fostering a robust inflammatory response that amplifies antitumor immunity (18). In parallel, OV infection generates pathogen-associated molecular patterns (PAMPs), and together with DAMPs, elicits strong innate immune recognition and activation through multiple pattern recognition receptors (PRRs), effectively breaking tumor-induced immune tolerance that limits conventional therapies (19, 20).

These effects converge on three critical immunoregulatory mechanisms that collectively shape the therapeutic efficacy of OV therapy:

(1) activation of the cyclic GMP-AMP synthase–stimulator of interferon genes (cGAS–STING) pathway,
(2) enhancement of dendritic cell-mediated antigen presentation and T-cell priming, and
(3) repolarization of tumor-associated macrophages (TAMs) toward a pro-inflammatory phenotype.

The cGAS–STING signaling axis represents a central mechanism of OV-induced immune activation. During viral replication, both viral double-stranded DNA and tumor-derived cytosolic DNA accumulate and are recognized by the DNA sensor cGAS, which catalyzes the synthesis of cyclic GMP-AMP (cGAMP) from ATP and GTP. cGAMP subsequently binds and activates STING (21, 22). Activated STING recruits TANK-binding kinase 1 (TBK1), leading to phosphorylation and nuclear translocation of IRF3, which directly drives type I interferon (IFN-I) expression. Concurrently, NF-κB signaling is engaged, resulting in robust production of pro-inflammatory cytokines such as TNF-α and IL-6 (21–23), thereby establishing an immune-inflamed tumor microenvironment that favors antitumor responses. However, due to the pronounced intratumoral heterogeneity of osteosarcoma, both the expression and functional activity of the cGAS–STING pathway vary considerably among tumor cells, resulting in differential production of inflammatory chemokines such as CXCL10 and CCL5 (24). These variations ultimately influence the efficiency of immune cell recruitment and the magnitude of antitumor immune activation (22).

Dendritic cell (DC)–mediated antigen presentation and T-cell priming serve as an essential intermediate link in the antitumor immune cascade triggered by oncolytic virotherapy. Upon OV infection, tumor-associated antigens (TAAs) and danger-associated molecular patterns (DAMPs) are released and rapidly recognized and internalized by DCs, thereby enabling efficient antigen presentation (25–27). Among these subsets, cDC1 plays a pivotal role by cross-presenting exogenous antigens on MHC-I molecules, resulting in the activation of CD8^+^ cytotoxic T lymphocytes (CTLs) and subsequent tumor cell killing (26–28). In addition, OV infection can directly upregulate MHC-I expression on tumor cells (19, 20), or indirectly enhance antigen presentation by activating the cGAS–STING pathway and promoting type I interferon production, which further increases the expression of co-stimulatory molecules (CD80, CD86) and MHC-I/MHC-II molecules on DCs (25–28), thereby helping overcome the impaired antigen presentation frequently observed in osteosarcoma (19, 20). Besides antigen presentation, co-stimulatory signaling, such as the CD40/CD40L axis, synergistically promotes the activation of CTLs and helper T cells. Together, the coordinated induction of antigen presentation and co-stimulation ultimately establishes a robust adaptive immune response network to exert antitumor immunity (29).

Reprogramming tumor-associated macrophages (TAMs) represents another critical component of OV-mediated remodeling of the tumor immune microenvironment. OV infection induces a local proinflammatory milieu and facilitates the repolarization of immunosuppressive M2-like TAMs into proinflammatory M1-like macrophages, thereby reshaping the tumor microenvironment (TME) (19, 30). Mechanistically, OVs suppress IL-4/IL-13–activated STAT6 signaling and IL-10–activated STAT3 signaling to reverse M2 polarization (31, 32). Meanwhile, DAMPs and pathogen-associated molecular patterns (PAMPs) released after OV infection activate TLR7/8/9 and trigger the MyD88–NF-κB signaling cascade, promoting STAT1 activation and driving the expression of M1-related genes including iNOS, TNF-α, and IL-12 (16, 31–35). This phenotypic transition is accompanied by a metabolic shift from oxidative phosphorylation typical of M2 macrophages toward a glycolytic program characteristic of M1 macrophages, which provides essential bioenergetic support for inflammatory responses and antitumor activity (23, 31). Furthermore, M2-to-M1 repolarization markedly enhances macrophage antigen-presenting capacity and phagocytic function (19, 30), thereby boosting NK cell and T-cell effector responses. This forms a positive feedback loop that sustains and amplifies antitumor immunity within the TME (19, 30).

OV-mediated immune activation follows a coordinated temporal cascade (25–28). In the early phase of infection, viral replication induces the rapid release of DAMPs and PAMPs (6, 8), which are sensed by tissue-resident DCs. Subsequently, activation of the cGAS–STING pathway drives type I interferon production (21–23), promoting DC maturation and upregulation of MHC-I and co-stimulatory molecules (25–28). Mature DCs then migrate to the draining lymph nodes, where they prime naïve CD8^+^ T cells via CD40–CD40L signaling (26–29). Ultimately, activated effector T cells home to the tumor site, where they cooperate with M1-polarized TAMs to execute cytotoxic activity (26–28).

**Figure 2:**
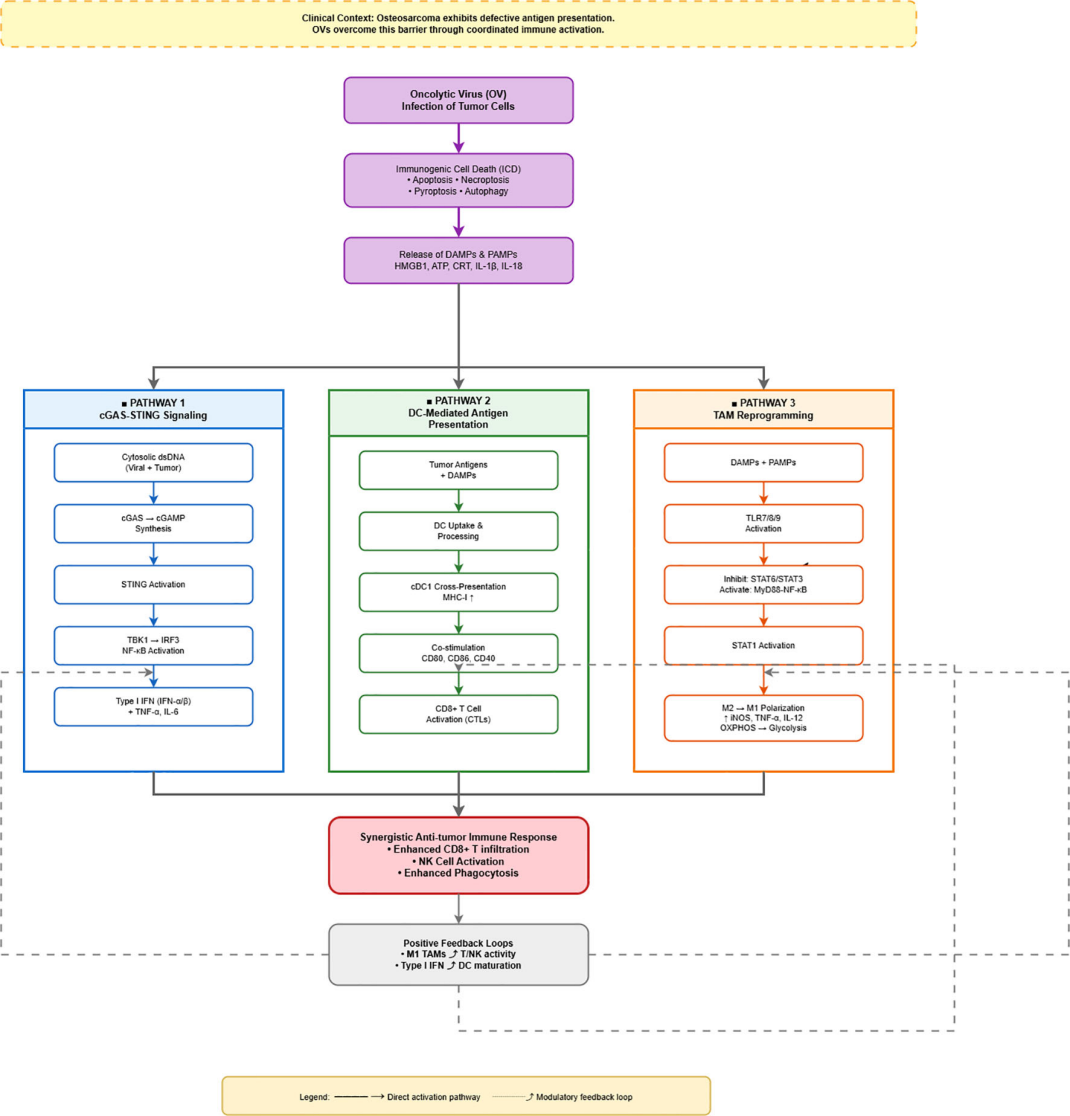
Mechanistic pathways of oncolytic virus-mediated antitumor immunity in osteosarcoma. This figure depicts how oncolytic viruses (OVs) overcome defective antigen presentation in osteosarcoma through coordinated immune activation. OV infection induces immunogenic cell death (ICD), releasing damage-associated molecular patterns (DAMPs) and pathogen-associated molecular patterns (PAMPs). These danger signals simultaneously activate three parallel pathways (color-coded columns): (1) cGAS-STING signaling (blue) recognizes cytosolic DNA and produces type I interferons and pro-inflammatory cytokines; (2) DC-mediated antigen presentation (green) enables cross-presentation via MHC-I and co-stimulation, activating CD8^+^ T cells; (3) TAM reprogramming (orange) activates TLR signaling to drive M2-to-M1 macrophage polarization with enhanced pro-inflammatory function. These pathways converge to generate a synergistic antitumor immune response characterized by enhanced T cell infiltration, NK cell activation, and increased phagocytosis. Positive feedback loops (dashed arrows) further amplify immunity: M1 TAMs enhance T/NK activity, while type I interferons promote DC maturation. Solid arrows indicate direct pathways; dashed arrows represent modulatory feedback. Color coding emphasizes the distinct yet coordinated nature of the three parallel immune activation mechanisms.

This spatiotemporally ordered process involves the coordinated engagement of three interdependent immune pathways (**Figure 2**): the cGAS–STING signaling axis (blue), DC-mediated antigen presentation and T-cell priming (green), and M2-to-M1 TAM repolarization (orange). These pathways reinforce each other through positive feedback loops, collectively driving a synergistic antitumor immune response. Detailed mechanisms, regulatory events, OV categories, and signaling interactions are systematically summarized in **Tables 1**-**4**: **Table 1** outlines OV-induced ICD molecular pathways; **Table 2** details cGAS–STING/type I IFN signaling; **Table 3** describes the molecular framework of the DC–T-cell activation axis; and **Table 4** compiles pathways and effectors involved in M2-to-M1 repolarization.

**Table 1:** Molecular mechanisms and signaling pathways of OV-induced immunogenic cell death (ICD) in osteosarcoma.

Mechanism	Key components/processes	Biological function	Applicable OV types	Ref.
DAMPs release	HMGB1, ATP, calreticulin (CRT)	Activates APC-mediated antigen presentation and promotes DC maturation	HSV, adenovirus, ORFV	(6, 8, 40, 60–64)
PAMPs release	Viral nucleic acids and proteins	Triggers PRRs, induces type I IFN signaling, enhances innate immunity	Broad OV species	(6, 8, 40, 62, 63)
Immunogenic apoptosis	Caspase-3/7	Tumor cell lysis and antigen release	HSV, adenovirus	(8, 60, 62, 63)
Programmed necrosis	MLKL	Immunogenic necroptosis with robust DAMPs release	HSV, SFV	(8, 62)
Pyroptosis	GSDME, caspase-1/8, IL-1β/18	Inflammatory rupture, promotes IL-1β release and T-cell infiltration	ORFV	(62, 65)
Autophagy-associated death	LC3, Beclin-1	Enhances antigen processing and improves T-cell priming	NDV	(62, 63)
Immune activation	Type I IFN, IL-1β, IL-18	Promotes DC activation and CTL responses	Multiple OV types	(8, 40, 60, 62–64)
Microenvironment remodeling	TAM reprogramming, NK/T cell activation	Converts “cold” tumors into immunologically “hot” tumors	HSV, GM-CSF-modified OVs	(6, 13, 40, 61)

This table summarizes seven major mechanistic modules of OV-induced ICD, including DAMP/PAMP release, pyroptosis, necroptosis, and autophagy pathways contributing cooperatively to antitumor immunity.

**Table 2:** cGAS–STING/type I interferon pathway activation by OVs in osteosarcoma.

Mechanism	Key molecules	Functional role	Influencing factors	Ref.
Cytosolic DNA accumulation	Viral or tumor-derived dsDNA	Activates cGAS as an innate immune sensor	OV infection or genomic instability	(21, 22, 66)
cGAS activation and cGAMP synthesis	cGAS, cGAMP	Produces second messenger cGAMP to activate STING	cGAS expression levels determine responsiveness	(21, 22)
STING activation and trafficking	STING, TBK1	Golgi translocation and TBK1 recruitment/activation	Cell-line-dependent signaling variations	(21, 22)
Downstream signaling	TBK1, IRF3	IRF3 phosphorylation and nuclear translocation to induce IFN genes	Pathway integrity determines IFN induction efficiency	(21, 22)
Effector cytokine production	IFN-α/β, TNF-α, IL-6	Enhances antitumor and antiviral signaling; activates DCs and T cells	Facilitates immune activation and cell recruitment	(21–23)
Immune microenvironment remodeling	M1 polarization, DAMPs	Suppresses immunosuppression and reshapes tumor microenvironment	Supports synergy with PD-1/PD-L1 blockade	(66, 67)
Cell line heterogeneity	STING, cGAS	Determines pathway activity and OV response	Biomarker for selecting responsive patients	(22)

This table outlines the full signaling cascade of OV-triggered cGAS–STING activation and emphasizes its bridging role in initiating antitumor immunity.

**Table 3:** OV-mediated activation of the DC–T cell axis in osteosarcoma.

Mechanism	Key elements	Functional consequences	Regulatory factors	Ref.
Tumor antigen release	TAAs, DAMPs	Initiates DC activation and priming	Critical ICD-associated step	(25–27)
DC activation and maturation	cGAS–STING, type I IFN, CD80/86, MHC-I/II	Enhances antigen presentation and T-cell priming	Predominantly dependent on Batf3-driven cDC1	(25–28)
Cross-presentation	cDC1, CD103^+^ DCs	Efficient activation of CD8^+^ T cells against neoantigens	Essential for immune memory	(26–28)
T-cell activation	CD8^+^ CTL, CD4^+^ Th, CD40/CD40L	Mediates tumor killing and long-term response	Requires DC-derived costimulation	(25–29)
Microenvironment remodeling	Reduced Tregs, increased CD8^+^/CD4^+^ infiltration	Limits immune escape and improves ICI response	OVs act as immune-sensitizing platforms	(26–28)

This table highlights key steps in DC–T cell crosstalk, including antigen presentation, T-cell activation, and microenvironment modulation.

**Table 4:** Molecular mechanisms and therapeutic strategies for TAM repolarization (M2 to M1).

Mechanism	Pathway/targets	Immunological effect	Representative agents	Ref.
Inhibition of M2 polarization	IL-4/IL-13-STAT6, IL-10-STAT3, TGF-β	Reduces pro-tumor functions and immunosuppression	STAT6/STAT3 inhibitors, anti-TGF-β	(31, 32, 68)
Activation of M1 polarization	TLR7/8/9, MyD88, NF-κB, STAT1	Enhances pro-inflammatory responses and antigen presentation	TLR agonists, CpG ODN, engineered exosomes	(16, 31–35, 69)
Metabolic reprogramming	PFKFB3, HK2	Supports glycolysis-driven M1 phenotype	2-DG, PFKFB3 inhibitors	(23, 31)
Targeting M2-specific markers	MARCO, IL4R, CD206	Eliminates or reprograms M2-like TAMs	Anti-MARCO Abs, IL4R inhibitors	(70, 71)
TME signaling regulation	DAMPs, PAMPs, IFN-γ, GM-CSF	Promotes M1 differentiation and immune recruitment	OV infection, GM-CSF-engineered cells	(16, 31–35, 72)
Suppression of immunosuppressive factors	IL-10, TGF-β, VEGF	Limits angiogenesis and immune escape	Neutralizing antibodies; small-molecule inhibitors	(16, 31–35, 72)

This table summarizes major signaling axes and therapeutic approaches for TAM repolarization, supporting TME remodeling strategies.

Despite compelling preclinical evidence suggesting that introducing immune checkpoint blockade after sufficient OV-induced antigen presentation may enhance T-cell effector function (11), this idealized temporal model may be disrupted in osteosarcoma due to multiple barriers. These include inefficient DC cross-presentation (26), excessive infiltration of immunosuppressive myeloid populations (12), and intrinsic STING pathway deficiencies within tumor cells (36). Importantly, these bottlenecks represent potential intervention targets for optimized combination strategies.

## 3 Opportunities

Oncolytic virotherapy has demonstrated promising antitumor efficacy in both preclinical and early clinical osteosarcoma studies (**Table 5**). In a phase I clinical trial involving pet dogs with naturally occurring osteosarcoma, VSV-IFNβ-NIS achieved long-term survival in approximately 35% of treated animals and markedly enhanced the expression of T-cell–related immune genes, indicating reinforced T-cell immunity and improved antitumor responses (37). Adenovirus-based platforms such as Ad5-Δ24-RGDOX have shown potent therapeutic activity, with tumor volume reductions of 30–60% in animal models and long-term survival extended to 35% in canine studies (12, 17). A novel armed OV, CAV2-AU-M3, demonstrated 40–70% decreases in cellular viability *in vitro* and was engineered to secrete functional anti-PD-1 antibodies, contributing to tumor progression control (14). In addition, combination strategies such as G-CSF plus adenovirus yielded further synergy, achieving tumor-inhibition rates of up to 70% (12).

**Table 5:** Summary of representative OVs, therapeutic efficacy, and safety in osteosarcoma.

OV type	Study model	Antitumor outcomes	Safety profile	Ref.
VSV-IFNβ-NIS	Canine phase I / spontaneous osteosarcoma	~35% survival improvement; enhanced T-cell response	Well tolerated; no severe toxicities	(37, 73)
Ad5-Δ24-RGDOX / ICOVIR-5	Phase I / animal models	Tumor regression 30–60%; canine long-term survival 35%	Mild–moderate fever; no DLT	(12, 17)
HSV-G207 / T-VEC + pembrolizumab	Preclinical / multi-tumor clinical trials	Increased CD8^+^ T-cell infiltration; improved ICI response	Favorable safety across tumor types	(6, 17)
Reovirus / CDV / H-1PV	Preclinical / animal studies	40–70% cancer cell viability reduction; significant tumor inhibition	High tolerability	(6, 17)
CAV2-AU-M3	*In vitro* / murine studies	40–70% tumor suppression; secretes anti-PD-1 antibody	No severe toxicity; high engineering potential	(14, 74)
G-CSF + adenovirus	Animal studies	Tumor inhibition 60–70%; increased TIL infiltration	No systemic toxicity observed	(12)
Trabectedin + HSV	Animal studies	Higher CR/PR rates versus monotherapy; synergistic inhibition	Good tolerability without increased toxicity	(10)

This table integrates key efficacy and safety evidence for OV monotherapy and combination strategies in osteosarcoma, covering canine clinical data, animal models, and *in vitro* studies.

Alongside these encouraging antitumor outcomes, OV therapy has also exhibited a favorable safety profile (**Table 5**). Across phase I clinical trials in both canines and humans, most adverse effects were limited to mild-to-moderate fever and local inflammatory reactions, without the onset of severe organ toxicities or dose-limiting toxicities (6, 12, 17). The phase I trial of Celyvir (ICOVIR-5/Δ24-RGD; NCT01844661) further confirmed the safety of adenoviral OVs in patients with advanced osteosarcoma, with a subset of individuals achieving disease stabilization or measurable tumor regression (12).

## 4 Challenges

### 4.1 Immunosuppressive tumor microenvironment and model limitations

The tumor microenvironment of osteosarcoma exhibits a strongly immunosuppressive profile, characterized by the predominance of M2-type macrophages, high levels of regulatory T cells (Tregs), excessive PD-L1 expression, and profoundly impaired T-cell and NK-cell functions. As a result, the antitumor efficacy of OV monotherapy is generally limited, with tumor inhibition rates typically below 20% (6, 38). In addition, commonly used immune-deficient and canine models fail to fully recapitulate the complexity of the human immune system, making it difficult to accurately reflect OV-induced immune responses and the dynamic evolution of the tumor microenvironment (6, 39). Sample sizes in these models are often small, which compromises statistical power (17).

### 4.2 Challenges in viral delivery and intratumoral distribution

Effective OV delivery and distribution within tumors face multiple barriers. During systemic administration, OVs are often neutralized by circulating antibodies, substantially reducing the viral load reaching the tumor site (12, 17). Osteosarcoma cells display significant heterogeneity in their expression of different OV-associated receptors, such as CAR and integrins αvβ3/5, leading to variable infection and lytic efficiency (17, 38). Moreover, the extracellular matrix (ECM) is structurally complex and highly heterogeneous; components such as collagen fibers and hyaluronic acid increase interstitial pressure and form physical barriers that hinder OV diffusion (40). Abnormal and irregular vascularization further limits drug perfusion in certain tumor regions, resulting in uneven viral distribution and suboptimal therapeutic concentrations (6, 41).

### 4.3 Safety, resistance, and neutralizing antibody responses

The therapeutic window for OVs is relatively narrow. Low-dose regimens often fail to achieve sufficient antitumor responses, whereas high-dose strategies are required for osteosarcoma cells with MDR1-mediated drug resistance, which may trigger systemic inflammatory reactions (10, 42). Additionally, high viral exposure leads to the induction of neutralizing antibodies (nAbs), impairing subsequent OV reinfection of tumor cells and significantly diminishing therapeutic efficacy upon repeated administration (43–46). Notably, pediatric patients are more susceptible to virus-associated immune toxicities, including hepatotoxicity and hematologic complications, necessitating special attention to treatment safety (47). These key challenges, their underlying mechanistic bases, and corresponding potential countermeasures are systematically summarized in **Table 6**.

**Table 6:** Key challenges, mechanistic basis, and potential countermeasures in oncolytic virotherapy for osteosarcoma.

Challenge	Mechanistic basis and evidence	Key findings	Strategies	Ref.
Immunosuppressive TME and limitations of preclinical models	High M2-type TAM and Treg infiltration; PD-L1 upregulation; impaired T/NK cell effector function; conventional immune-deficient models fail to recapitulate human immunity	OV monotherapy yields <20% tumor inhibition; combination therapies elevate inhibition to 60–70%; immune-deficient models poorly represent OV-induced immune dynamics	Combine with PD-1/PD-L1 blockade, GM-CSF/G-CSF; develop humanized immune mouse models; expand studies using spontaneous canine osteosarcoma	(6, 13, 17, 38, 39, 48)
Limited OV delivery and intratumoral distribution	Receptor heterogeneity (e.g., CAR, integrins); ECM barriers (collagen, hyaluronic acid); abnormal vasculature restricts perfusion; dense bone matrix limits penetration	Intratumoral or MSC-based OV delivery increases viral titers 2–5× compared to systemic dosing; necrotic tumor cores remain poorly reached	Personalized OV serotype selection; co-expression of hyaluronidase/collagenase; optimized MSC carriers (chemotaxis engineering, immune-shielding); bone-targeted delivery platforms	(6, 12, 17, 38, 40, 41)
Safety concerns, resistance, and neutralizing antibody (nAb) responses	High-dose OVs trigger systemic inflammation/cytokine release; MDR1 upregulation causes viro-resistance; rapid nAb synthesis clears OVs; pediatric patients are more susceptible to toxicity	High-dose regimens induce systemic inflammatory reactions; >50% efficacy loss upon repeat dosing; pediatric hepatotoxicity incidence higher than adults	Serotype-switching strategies; liposomal/polymer encapsulation to evade nAbs; low-dose immunomodulators to balance immune activation; optimized PK/PD-guided dosing schedules	(10, 42, 43, 46, 47)

This table summarizes three major barriers in OV therapy for osteosarcoma—TME-induced immune suppression, delivery constraints, and safety/resistance issues—and highlights corresponding mechanistic evidence and counteracting strategies.

## 5 Discussion

To address immunosuppressive tumor microenvironmental barriers, the next 5–10 years will likely see increasing use of combination therapies including PD-1 inhibitors to restore T-cell function, potentially improving tumor inhibition rates from <20% with OV monotherapy to 60–70% (6, 38). The coordinated administration of G-CSF or GM-CSF is expected to enhance tumor-infiltrating lymphocyte (TIL) recruitment and functionality (12, 13). Furthermore, targeting macrophage polarization using anti-M2 agents and M1-promoting cytokines may convert the immunologically “cold” tumor phenotype into a “hot” state, thereby strengthening NK- and T-cell-mediated antitumor responses (48). AI-based prediction models will likely support personalized OV selection and virus–host matching to overcome current therapeutic limitations and significantly improve response rates (49–52).

Advances in systemic delivery and intratumoral distribution are anticipated with the development of multiple novel strategies. Mesenchymal stem cell (MSC)-based carrier systems show high promise by shielding OVs from nAb-mediated clearance and exploiting tumor-homing properties to increase intratumoral viral accumulation by 2–5-fold (12, 17). Local intratumoral administration will further improve viral concentration at target sites (53). Viral engineering approaches, including the insertion of RGD motifs to enhance receptor binding and the overexpression of ECM-degrading enzymes such as hyaluronidase, are expected to facilitate OV penetration through dense matrix structures (6). Additionally, smart nanocarriers combined with AI-guided real-time monitoring will allow dynamic adjustment of treatment strategies and optimization of long-term survival outcomes (54–56).

To mitigate risks of toxicity and viral resistance, future approaches will incorporate AI-assisted dose optimization to maximize efficacy while minimizing systemic inflammatory responses (42). Combination regimens such as OBP-702 with chemotherapy may suppress MDR1-mediated resistance and reduce reliance on high-dose OVs, adding 30–40% tumor suppression benefit (10). AI-based antibody kinetics modeling will aid in optimizing dosing intervals and minimizing nAb accumulation (43, 47), while serotype rotation or liposomal shielding will extend therapeutic windows (46, 57). For pediatric patients, individualized toxicity monitoring supported by AI may help balance efficacy and safety more precisely (45).

Additionally, in cases of skeletal metastases of unknown primary (SMUP) coexisting with osteosarcoma, both diseases may exhibit RANK/RANKL pathway dysregulation and increased bone turnover markers such as CTX and alkaline phosphatase. These findings indicate shared pathophysiological mechanisms in the bone microenvironment that modulate OV diffusion, replication, and immune activation (58). Thus, further mechanistic studies incorporating AI-assisted platforms may accelerate the translation of OV-based precision therapies and enable coordinated management of such comorbid conditions (59).

## Data Availability

The original contributions presented in the study are included in the article/supplementary material. Further inquiries can be directed to the corresponding author.
